# The Summer Undergraduate Research Experience as a Work-Integrated Learning Opportunity and Potential Pathway to Publication in Psychology

**DOI:** 10.3389/fpsyg.2019.00541

**Published:** 2019-03-20

**Authors:** Raelee M. Golding, Lauren J. Breen, Amanda E. Krause, Peter J. Allen

**Affiliations:** ^1^ School of Psychology, Curtin University, Perth, WA, Australia; ^2^ Melbourne Conservatorium of Music, University of Melbourne, Melbourne, VIC, Australia; ^3^ School of Psychological Science, University of Bristol, Bristol, United Kingdom

**Keywords:** undergraduate research experience, work-integrated learning, psychology, undergraduate publication, research supervision

## Abstract

Unlike disciplines which focus on skill development from year one of a bachelor’s degree, training in psychology in Australia follows the scientist-practitioner model. According to this model, an undergraduate psychology degree should focus on the scientific principles underpinning the discipline and provide a foundation for the development of professional skills in graduate school. However, most Australian psychology undergraduates do not continue into graduate school, and concerns have been raised about their lack of applied skills and work-readiness. Work-integrated learning (WIL) refers to strategies aimed at providing students with practical experiences (e.g., fieldwork, placements, and internships) directly related to their course of study. The objective of WIL is to increase work-readiness. Accreditation standards coupled with the norms of the discipline have historically prevented the inclusion of typical WIL experiences in Australian undergraduate psychology degrees. However, one particular type of WIL activity—the undergraduate research experience (URE)—is particularly suited to psychology. In a typical URE, students collaborate with faculty to conduct research designed to make an original contribution to their field. The current study is a qualitative investigation of stakeholder perceptions of a competitive summer URE program ran from 2012 to 2016. Six faculty members and seven undergraduate students were engaged in semi-structured interviews about their URE experiences. Constructed themes broadly reflected the benefits and challenges of the program and included *work-readiness and additional research experience, networking and teamwork, publication, quality of experience* and *equity* of opportunities. Faculty members and students spoke favorably of their UREs in most cases, although issues of administration and financial concerns were mentioned consistently, as were concerns about the length, timing, and nature of projects. Students reported skill development and networking as two of the key benefits of their participation in the program, and article publication was seen as particularly beneficial to career prospects. Our findings suggest that student co-authored publications resulting from UREs are possible, but careful thought is required to optimize their likelihood. Overall, this research adds to a growing literature suggesting that UREs can confer a range of benefits to Australian psychology schools related to increased research capacity and student satisfaction.

## Introduction

Work-integrated learning (WIL) refers to “approaches and strategies to integrate theory with the practice of work within a purposefully designed curriculum” (Patrick et al., 2009, p. v). The primary objective of WIL is to improve graduate employability by providing students with practical experiences that are directly related to their course of study (Smith et al., 2014; Universities Australia et al., 2015). WIL activities are diverse and commonly include fieldwork, placements, practicums, and internships. Such WIL activities are routinely embedded in many undergraduate degree courses, particularly those that graduate students “ready” for entry-level practice in their chosen professions (e.g., nursing, accounting, engineering, architecture, among many others). Research suggests that opportunities to engage in and/or facilitate such activities are associated with a range of positive outcomes for students (Dressler and Keeling, 2011), employers (Braunstein et al., 2011), and education providers (Crump and Johnsson, 2011).

Unlike disciplines which focus on the development of specialized skills from the first year of the bachelor’s degree, training in psychology in Australia follows the “scientist-practitioner” model (Lipp et al., 2007; Provost et al., 2010). According to this model, a four-year undergraduate psychology degree should focus on the scientific principles underpinning the discipline and the development of generic competencies, such as the ability to conduct valid research, think critically, behave ethically, communicate effectively, and demonstrate psychological literacy (Cranney et al., 2009; Provost et al., 2010). These competencies position graduates for employment in a range of industries (Appleby, 2018; Halonen and Dunn, 2018), though not for immediate employment as a registered psychologist (Littlefield, 2016). Most Australian psychology undergraduates do not continue into graduate school, which is the key pathway for employment as a psychologist (Hamilton et al., 2018). The absence of practicum/placement opportunities is the most frequent criticism of Australian undergraduate psychology courses, and psychology graduates tend to report that they lack professional and practical skills after completing a bachelor’s degree (Martin et al., 2013). Furthermore, employers and graduate placement supervisors often comment on the limited practical skills of four-year graduates (Breen et al., 2003; Pachana et al., 2011; Sheen et al., 2015).

Although there are moves toward increasing specialized skill training in the final year of the bachelor’s degree (see the pre-professional competences specified in the Australian Psychology Accreditation Council’s [APAC], 2018, new standards, which became effective in January 2019), historical accreditation standards (APAC, 2010) combined with the norms of the discipline have precluded the inclusion of most typical WIL experiences in Australian undergraduate psychology degrees. However, one particular type of WIL activity—the undergraduate research experience (URE)—seems particularly suited to psychology.

In a typical URE, a student will collaborate (either individually, or as part of a small team) with a professional researcher/faculty member to conduct a piece of research designed to make an original intellectual contribution to their discipline (National Conferences on Undergraduate Research and the Council on Undergraduate Research, 2005). UREs can be considered as apprenticeships (Zimbardi and Myatt, 2014) and a form of professional socialization (Hunter et al., 2007), and research indicates that they afford students a range of benefits. For example, Kardash (2000) observed that science undergraduates self-reported significant gains in reading and using primary literature, formulating hypotheses, conducing data analysis, and interpreting and communicating scientific findings following URE participation. Liberal arts college students self-reported similar skill development, numerous personal/professional gains (e.g., increased confidence in their ability to conduct research, contribute to science, establish collegial relationships etc.), and an increased capacity to think and work like a scientist (Seymour et al., 2004). In a large study comparing alumni who had engaged in UREs with alumni matched on a range of personal characteristics, but without such experiences, Bauer and Bennett (2003) observed similar effects. Those alumni who had participated in UREs self-reported a greater ability to apply and understand scientific findings, think logically, solve problems creatively, use statistics and information technologies, adapt to change, critically consume research literature, work independently, and communicate effectively. The alumni who had participated in UREs also self-reported greater overall satisfaction with their college/university experience and were more likely to have subsequently enrolled in graduate studies. The magnitude of the effects observed by Bauer and Bennett (2003) appeared to increase commensurate with the length/intensity of the URE.

Multiple points of interest emerge when reviewing the URE literature. First, it has overwhelmingly originated from the United States, where a large national infrastructure has developed to fund and support UREs. For example, the National Science Foundation alone typically budgets over US$70 million per annum for their research experiences for undergraduates program (McDevitt et al., 2017). No comparable infrastructure exists in Australia. Second, many of the skills gained from a URE (e.g., the ability to conduct valid research, communicate effectively, behave professionally, etc.) map closely onto the graduate attributes for the four-year Australian undergraduate psychology degree articulated by Cranney et al. (2009) and subsequently endorsed by APAC (2010). Third, UREs have the potential to provide meaningful WIL experiences in Australian undergraduate psychology courses, where more traditional forms of WIL are difficult, or impossible. Increasing the WIL opportunities afforded to Australian university students has been identified as a priority by both government and industry bodies (Universities Australia et al., 2015). Despite these points, no research that we are aware of has focused on a URE as a potential WIL opportunity from the perspectives of Australian psychology undergraduates and faculty members. The first purpose of the current research was to redress this deficit. Additionally, given the established potential of UREs to contribute to knowledge generation and dissemination, we also wanted to explore the potential for UREs to lead to publication opportunities for undergraduate students in psychology.

## Method

### Context

The School of Psychology at Curtin University initiated an annual “summer scholarship” URE program in 2012, which ran until 2016 when budgetary constraints forced its cancellation. The program allowed students to work closely with one or more faculty members on a project of mutual interest. Each student was paid AU$2,000 for up to 30 days’ work. During that time, students completed a variety of tasks oriented toward progressing the project (e.g., participant recruitment, data collection, data analysis and interpretation, reviewing and synthesizing literature, report writing, and dissemination). The URE scholarship program was intended to give students applied research experience prior to their third year of study.

Participation in the program was competitive for both faculty and students. Faculty members proposed projects that were evaluated according to their feasibility and the quality and variety of opportunities they were likely to afford students. Student applicants were selected based on their prior academic performance. Student applications numbered approximately 40–50 per year, and up to seven scholarships were awarded annually. Each participating faculty participant was asked to complete a brief written report on the project following the completion of the scholarship.

### Research Design

We addressed our research question qualitatively. This afforded us opportunities to explore and understand the meaning and value (or otherwise) of an understudied phenomenon, an Australian psychology URE program, from the perspectives of key stakeholders. We adopted the epistemological position of critical realism (Forrester, 2010). That is, we recognize that knowledge is constructed and context bound, and that research is a social process. However, this process can provide (imperfect) access to an authentic reality that exists beyond our methods. Consequently, critical realism can be seen as taking a middle ground between social constructionism and extreme positivism, allowing for rigor and reflexivity concurrently (Forrester, 2010). Semi-structured interviews were conducted and thematically analyzed (Braun and Clarke, 2006). These data were complemented with archival records (including faculty members’ written reports) of the longer-term outcomes of scholarship projects, where available.

### Participants

Between 2012 and 2016, 22 psychology students and 16 faculty member supervisors were involved in the URE program. Following ethics approval (reference: HRE2017-0380), each of these stakeholders (with the exception of two authors who previously supervised UREs and two supervisors no longer employed by the university) was emailed an invitation to participate in the study by a school administrator independent of the research team. Seven students and six faculty members (total *N* = 13) responded to this invitation and were subsequently interviewed for this study. Consequently, our response rate was 34%. Gender descriptions have been purposefully withheld from this article to assure participant confidentiality (Kaiser, 2009). It was estimated that approximately 12 interviews would result in saturation (Ryan et al., 2007), although it is recognized that the concept of saturation is contentious (O’Reilly and Parker, 2013), particularly when multiple stakeholder perspectives are being considered. In our case, we believe that saturation at the theme level was reached (Guest et al., 2006), and this determination was informed by the concept of information power (Malterud et al., 2015).

### Materials

The literature review informed the development of separate semi-structured interview protocols for student and faculty member participants. The student protocol included questions about the nature of the URE experience and if/how it has informed subsequent academic and professional development. The faculty protocol included questions about the experience of supervising projects and the role that UREs can play in the undergraduate curriculum and employment preparation. Both protocols are included in our Supplementary Material.

### Procedure

Individual interviews were scheduled at mutually convenient times and locations with all of the students and faculty members who responded to our call for participants. All interviews were conducted by the first author, lasted between 18 and 42 min (*M* = 33 min), and were audio recorded with the consent of participants. They were semi-structured in the sense that all the main questions on the interview protocols were asked of participants, although not necessarily in the same order. Furthermore, the number and nature of prompts used varied according to participants’ responses. Such flexibility allowed for the development of rapport and exploration of issues as they were raised by participants, which explains the variability in interview length. The interview recordings were transcribed verbatim before being securely erased.

### Data Analysis

The interview transcripts were imported into NVivo for data management and analysis. The transcripts were thematically analyzed following the six-step procedure described by Braun and Clarke (2006). This entailed (a) initial familiarization with the data, (b) line-by-line coding, (c) collation of recurrent codes into potential themes, (d) theme checking throughout single transcripts, followed by the entire dataset, (e) creation of labels and definitions for emergent themes, and (f) utilization of themes and representative quotes to construct findings addressing the aims of the study (Braun and Clarke, 2006). The first author led the analysis, whilst the remaining authors contributed to peer coding and provided regular feedback on emergent themes and the development of the analysis. Names and pseudonyms were avoided in favor of gender-neutral pronouns (e.g., their) in the preparation of this manuscript to increase the confidentiality of interviewees who are known to each other and are therefore potentially identifiable (Kaiser, 2009). These decisions afforded participant anonymity beyond the research team. Themes and selected data extracts were drawn from the whole data corpus to reflect the entire sample. Data extracts appear with a code indicating faculty (F1–6) or student (S1–7) status. Journaling and memo writing were used as an audit trail to track research decisions (Forrester, 2010). The memos included daily research activity lists, queries, thoughts, anticipated categories, data issues, collation of earlier memos, summaries of meetings, and reflections.

## Findings

The constructed themes were grouped broadly as benefits or challenges. Benefits included *work-readiness and additional research experience*, *networking and teamwork*, and *publication*. All participants were also critical about aspects of the program, highlighting particular challenges with the program. Notably, themes concerning the *quality of the experience* and the *equity* of the program highlight these challenges.

### Work-Readiness and Additional Research Experience

Most participants (*n* = 11) described their involvement in the URE positively, with both faculty and students reporting a range of benefits associated with perceived work-readiness. The URE was described as positive, enriching, advantageous to future endeavors and employment options, and an opportunity to foster professional connections that are not easily made via the usual undergraduate course-based experiences.

Within the URE context, there were benefits attained via participation in a new research-based experience that might simulate skill development and thus help prepare students for future employment opportunities: “*In my experience, they are greatly beneficial because they give students an opportunity to do things that otherwise, they wouldn’t be able to*” (F2). Here, the faculty member aptly summarizes a benefit of the URE opportunity, also synonymous with exposure to new experiences, such as tutoring positions, research assistant employment, and national or international networking via project involvement, conference presentations, and article publication, all of which were outcomes described in interviews with students.

*It seemed like a pretty great opportunity to further my skills, and so I only could see the benefit in that. I was really really set on quantitative research at the time, as well, and so I really wanted to be professional more than proficient at it. I thought it would be really beneficial.* (S4)

Here, we see the enthusiasm that URE participation evokes in students whose experiences were positive and productive.

Related to, or possibly because of, developing research skills, the URE program enhanced perceived employability and even created future opportunities for research experiences. Four of the seven student interviewees reported securing employment as research assistants as a direct consequence of their involvement in the URE.

*Afterwards they asked me to come back and do a couple more things, but as paid work. I did that for about half a year.* (S2)

*I just continued working as a research assistant the whole time, so that was good and very flexible. So during semester I didn’t do very many hours and then I did a lot of hours over summer, so that was good.* (S1)

This suggests that the skill development that occurs in a URE many promote student employability, at least in research contexts. Two further cases were mentioned by faculty. For instance, one faculty member said *“I think it’s a really, really good opportunity for the students. It becomes a CV* [curriculum vitae] *item; we work towards getting them some kind of output* [i.e., publication or conference presentation] *that they can list as well as the experience”* (F5). In this way, program participation was perceived to bolster work-readiness skills prior to graduation, where these types of skills will be essential for career success.

### Networking and Teamwork

The URE was seen by many participants as a platform for initiating and developing professional relationships. This theme was evident in all student interviews and five of the six faculty interviews. Faculty described the URE as an opportunity to recruit future research (e.g., honors and graduate) students. For example, one faculty member described how their role did not include regular undergraduate teaching, and thus their ability to meet and capture the interest of prospective research students was limited. This faculty member explained that many students select honors and graduate projects based on who they know, rather than what they will be researching. Thus, involvement in the URE program provided an important opportunity to achieve some name, face, and research area recognition:

*It’s a networking opportunity. The first thing is to give a student who is interested in research an opportunity to do some, and get their hands dirty and see what it means to actually work in a lab … and then hopefully interest them enough, or sufficiently in the stuff that we’re doing so that they may come back to do honours, or further [graduate research].* (F2)

The URE can be a microcosm of later, more substantial research experiences, and an environment that can foster relationships that may continue well beyond the summer. This is reflected in student perspectives, with one student explaining:

*The best thing to come out of it was the relationship I built with my supervisor, who then went on to be my supervisor for honors and now graduate [studies] … the people I worked with in the team. That was probably the best experience, and what I was hoping for.* (S1)

It appears that, when decisions about future study are made, subject matter can play a secondary role to personal relationships. At least some students make decisions about future study based primarily on who they know, which can make it difficult for faculty without undergraduate teaching responsibilities to attract capable research students. UREs can provide a valuable recruitment vehicle for such faculty.

Students who participated in projects that were situated in research labs or teams appeared to have particularly valued the experience of teamwork. For example, “*I really learned about the value of working together. I mean, I already believed in it, but I just saw what that team achieved by working together*” (S4). Equally, faculty members described the value of supervising in a team, which ensured that students were appropriately supported, even during very busy times.

*The students were able to come in and sit with us. They were with us nine-to-five, five days a week. They got to hear what was going on. So if I couldn’t help them immediately when they had a query, there were all my postdocs around who could give them assistance. And I made sure they were part of teams so there were other people always working on whatever they were working on as well.* (F5)

### Publication

For faculty, the key motivation behind applying for a scholarship student was usually the prospect of progressing a specific piece of research. *“We got a rather wonderful student who then worked with us on a systematic review…”* (F1). This meant that they designed the scholarship opportunity to lead to eventual publication. In such cases, the URE often helped faculty achieve this goal. *“This paper [would have been] impossible without this scholarship in the beginning … [it] made that paper possible”* (F3). However, a completed manuscript for publication was not a program guarantee. For example, just 8 of the 24 projects that we were able to locate records for led to published papers (with a ninth about to be submitted). Faculty generally acknowledged that although undergraduate students can contribute to the work involved in publishing research, the majority of it is beyond their level of ability and expertise.

*When the student finished, there was still an awful lot of work to do…* (F1)

*It really is dependent on you having the kind of data that someone at that level can work with. They don’t have the skills yet to write it up in a way that could be immediately publishable, or even in a report… Both those students were good though, but it still takes reworking.* (F6)

This may account for why, of the eight papers we identified, only three were co-authored by students. However, the contributions of the relevant students were acknowledged on most of the rest.

For students, the prospect of publication did not drive their decision to apply for a scholarship. However, for some, it was a favorable consequence of their involvement with the program.

*She’d always also mentioned that this could turn into a publication for me as well… So I was like "yeah, that sounds like a great opportunity”.* (S5)

*…I went straight into data cleaning and screening … and then we went into the analysis. From there, we ended up writing up a paper. Well, we wrote up a draft and then our supervisor made changes to it. From there, [the supervisor] submitted it for publication. I think not long ago we got approved, so that was really cool!* (S3)

S3 described in detail how they were involved in multiple steps of the publication process, from initial journal selection, through rejections, revisions, and acceptance. However, the other students were not so involved. Some lost contact with their work at the conclusion of their URE, and others did not contribute sufficiently to justify co-authorship, or were involved in a project that supervisors ultimately decided not to pursue.

*Maybe some of the data analysis was used, but I don’t think my name was actually put on any papers because I probably would have been notified*. (S2)

*Nothing’s happened in five months. Maybe five months ago they decided it was all a waste of time and they weren’t going to do it anymore and that was the end of it. Maybe I’ll look in a couple years and search my name in one of the databases and see if my name pops up somewhere.* (S7)

Finally, the opportunity to co-author a conference presentation served as satisfactory outcome for some students:

S*o from that then the opportunity came to speak at a conference with this paper, cause it was rejected for publications*. (S1)

### Quality of Experience

Although most faculty and students described the URE in positive terms, challenges and criticisms of the program were also expressed by every student to varying degrees. In these cases, the challenges often stemmed from the reality of the program not meeting students’ and faculty members’ expectations. This theme, *quality of experience*, concerned issues around administrative matters, whether expectations were met, and the quality of the supervision and learning opportunities.

*My expectation was that I would get access to people and that it would be a collegiate kind of environment. It would be an exciting practical experience in research, so it would give me some skills that I hadn’t been exposed to before and that it was an opportunity to develop some relationships with strictly academic staff. I guess, truth be told, none of that really happened.* (S7)

One student reported that their experience was tarnished by issues concerning administration, remuneration, and isolation throughout the project. Although the scheme was advertised as a “scholarship,” the scholarship holders were paid as employees of the university, with their pay subject to income tax. For many, this meant that their $2,000 scholarship was worth around $1,600 (with the remaining $400 withheld to offset their potential tax liability at the end of the financial year). Additionally, students were not typically assigned a workspace and, therefore, completed much of their work away from campus and the project supervisor/research teams.

Also linked to the administration of the URE was a desire on the part of students to have more input into the nature of their experience:

*It certainly wasn’t a project that I feel like I would have chosen if I’d known a lot of detail about it. I just went in blind and heard about it when I got the notification that I got in. Then learned who I was going to be working with, so no real choice about any of that stuff.* (S7)

A further common criticism of the program was that experiences tended to fall within skill areas that students felt they had already developed, such as literature searches, rather than providing opportunities for new experiences. Additionally, students and faculty at times were frustrated that the quality of the learning experience was not optimized, meaning that at least some URE projects were an under-utilized opportunity. For example, one student was assigned to a project that did not yet have ethics approval, which reduced the availability of tasks and learning opportunities and resulted in the student mainly practicing skills they felt were already well developed:

*It could have been way better. I could have been used better. I could have learned more than the same old things. I mean through the degree obviously we already do a lot of finding articles. And Googling isn’t anything revolutionary. It’s quite boring.* (S6)

This sense of mismatched expectations was echoed by some faculty, suggesting that a more rigorous matching procedure could potentially offer more value to the experiences of all involved parties.

*…a better marrying up of us knowing what they want to do and them knowing what we want them to do and seeing where we can find parallels, but hopefully with them being prepared to just ‘suck it and see’ a little bit as well.* (F5)

Faculty also felt that their ability to provide rich learning experiences for students was limited by the capabilities and knowledge of students within the context of available projects and datasets.

*Maybe whether there’s some sort of a matching scheme between the skills students have and the projects was possible, but again, given how limited the students are in where they are in their career … maybe not. It’s hard to tell, but maybe a little bit more background on the student so that we can say, okay, they have done this unit or they haven’t done this unit or they’re terrible at stats. Maybe we would reconsider or do a qualitative component. As I said, with the student we had, that wasn’t an issue, but I could see there being a potential mismatch there where your strengths are in one thing, but you’re put onto another project because you like the sound of it. Actually sometimes it’s not the topic area that’s important so much as the research skills that you’ve got.* (F1)

Another faculty member found the program disappointing when assigned a student who was unable to complete assigned tasks: “*I was expecting commitment. I always had commitment, and I didn’t get any commitment. It was disappointing, but I just thought, well that’s life, but I’m not doing another one*” (F6). Faculty members clearly acknowledged that the quality of the experience was contingent on how well people, topics, work ethic, and skills aligned.

Student and faculty experiences of un-met and mismatched expectations speak to the need for quality controls that ensure URE projects provide a diversity of opportunities for student participants as well as those providing supervision. A faculty member spoke passionately about their love of supervision, and the potential to shape the next generation, with insightful observations about the quality of the experience being so important:

*Making sure that supervisors don’t see it as just cheap labour. There’s a risk that even with honours projects, some supervisors can be seen to be putting their [own] needs first. They might not necessarily be, but the students can sometimes feel like they are a research assistant, not doing a research project.* (F1)

Although there was an acknowledged framework for the research component of the URE, there did not appear to be a framework in place to monitor the quality of supervision, as evidenced when F1 was probed further:

*When you put the proposal in, there’s rules as to what a proposal has to do, and so there’s clear-ish guidelines about [how] it has to be a learning experience and it has to fulfil certain criteria. Regarding the skills the student has to obtain, there are rules. Regarding how to manage the relationship with the supervisor, there’s nothing that I’m aware of.* (F1)

Beyond relying on the teaching experiences of each supervisor, the experiences of the students in our study suggested a need for explicit guidance to supervisors around facilitating a diversity of learning opportunities for students, as well as ongoing oversight to ensure these guidelines are followed.

*It was quite useful because essentially, from a selfish perspective, it enabled me to get some research assistant work without paying because the school was covering the cost. Because the students volunteered … they were quite motivated.* (F4)

### Equity

This theme was evident in the interviews with two students and three faculty members. It refers to the extent to which the URE was accessible to all students, versus only relatively affluent high achievers. Obviously, when there is competition for limited places, a selection process is required. This theme questions the extent to which this selection process should be purely grades-based and disregard qualities like motivation and diligence. For example, one student said:

*The individuals who get certain grades, or pass a certain grade point, are given set opportunities that I don’t know are experienced by everyone. So, what if someone really did want to further their quantitative skills and do some research assistant work for a scholarship program, but they couldn’t because their grades were not high enough, or something like that? And so, I just wonder about how this shapes individuals’ opportunities. The people who are doing well get more opportunities, and the people who didn’t get it the first time have less opportunities, and how that might perpetuate certain expectations.* (S5)

Here, we can see an altruistic concern from a student of high ability toward peers, and recognition of the notion that unless all students have equal access to opportunities like this URE program, the best students are set up to succeed in future endeavors, while more average students remain where they are. This perspective sets up an interesting dichotomy between faculty perceptions of student ability as being quite limited when only having completed half the degree, while successful applicants to the program are all high achievers, and considered more skilled than the general psychology cohort. From another faculty member, a similar although more economically driven equity criticism was raised:

*The difficulty with the program as it stands is it probably excludes a lot of our other students because they can’t give up a month because they’re working and so on and so forth. Again, a part-time thing might open it up to [all] students rather than the better off who are able to say, “All right, I’ll give up my time for a month and I don’t have to go and work in [a supermarket] or whatever”. I think for the learning experience but also in terms of equity, maybe part-time over a little bit of a longer period might work. The student has to balance that against work and so on.* (F1)

The point about running the URE less intensively over a longer period of time was raised by several faculty members (*n* = 4) and students (*n* = 4). Concerns were also raised about its timing over the summer tuition free break, when faculty tend to take annual leave, and because the subject pool is only operational during semesters, recruiting participants for many research projects is challenging.

## Discussion

The aim of this qualitative study was to illuminate the experiences of supervisors and students involved in an Australian summer URE. We were particularly interested in understanding the URE as a potential WIL opportunity from the perspectives of Australian psychology undergraduates and faculty members and to explore the potential for UREs to lead to publication opportunities for undergraduate students in psychology. Overall, the URE was a positive and engaging experience for most of the faculty and student participants interviewed in that it (1) exposed students to the research process and helped them develop skills and opportunities for additional research opportunities during and immediately after their undergraduate studies; (2) enabled networking, teamwork, and mentoring opportunities; (3) provided some students with the opportunity to work in research teams and labs; and (4) engaged some students in publishable research, through journal article and conference presentation co-authorship.

Although progressing publishable research was a higher priority for faculty than students when applying for one of the competitive URE scholarships, both benefitted when a scholarship project ended up in print. Our interviews suggested that, although by no means guaranteed, this happened with some degree of regularity. So far, 8 of the 24 projects led to a published paper. In light of the strict requirements for authorship (National Health and Medical Research Council, 2018), students co-authored three of these (Roberts and Rajah-Kanagasabai, 2013; Allen et al., 2016, 2017), though their contributions were acknowledged on most of the rest.

Four of the seven student participants described further employment in research-related roles to be a result of their URE, which suggests that UREs may confer advantages vis-a-vis work-readiness and employability. UREs are a way for educators to help students build confidence and self-efficacy (Hamilton et al., 2018). In a study on the benefits of conference presentations, something experienced by two student participants in our study, 53% of students described improvement in their ability to perform well on various tasks and 41% of students cited increased confidence about transitioning to the workplace (Freudenberg et al., 2008). Work readiness and graduate employability are currently priority areas in Australian higher education and are likely to have future funding implications. UREs can help universities address these priority areas (Hamilton et al., 2018). Further, although not all students in the current study had clear career goals, the URE provided an opportunity to consider the possibility of a research career. For others, the URE prompted greater commitment to nonresearch career pathways. Either way, exposure to UREs can help focus undergraduate students on career possibilities, contribute to development of their professional identities, and consequently enhance their employability (Cranney et al., 2008; Nyström et al., 2008).

Undergraduate psychology degrees train generic skills, including collaboration, communication, and problem solving, that are valued by employers in a range of industries (Halonen and Dunn, 2018; Hamilton et al., 2018). Further, the benefits of interdisciplinary collaborations are becoming more widely recognized as necessary in undergraduate psychology degrees, to mirror the multidisciplinary nature of many workplaces (Cranney and Dunn, 2011). Consequently, an ability to demonstrate success in team environments, such as those provided in UREs, can be an advantage to psychology graduates (Hamilton et al., 2018). Teamwork within UREs enables students to seek advice from multiple colleagues and supervisors and faculty to juggle demanding schedules more easily. This structure is likely to reflect the realities of many workplaces (Cranney, 2013). This appeared to be the case in our study, although the benefits of teamwork and networking and were unevenly spread across student participants.

Equity was a recurring theme for faculty and students, with two perspectives linked to student ability and student affluence. Grades have been the leading indicator of success in academia; however, research is beginning to question how well grades translate to success and capability in the workplace (Hamilton et al., 2018). One Australian study suggested that 60% of graduates felt advantaged by participation in WIL and 74% of graduates saw a relationship between the WIL and their career trajectory (Crebert et al., 2004). In the specific context of undergraduate psychology in Australia, it has been suggested that exposure to WIL and UREs too early, or in conditions where the student does not have a positive experience, can negatively impact confidence, course engagement, and the motivation to conduct future research (Hamilton et al., 2018). Access to these opportunities needs to be delivered in a way that is less grade-reliant and more sensitive to individual circumstances. An interview process is one possibility here (Hamilton et al., 2018) and could have the additional benefit of aiding the match between students, faculty, and projects. However, the feasbility of this process would depend on adequate resourcing.

The URE was not immune to criticism, and the experiences of students and faculty were not universally positive. Participants provided more detail in describing challenges, and this is reflected in the length of extracts reported in our findings section. Reflecting on their views regarding the program’s shortcomings can provide suggestions for the future development and administration of psychology UREs. This is most clearly illustrated when considering whether the URE opportunity met the expectations of faculty and students. The discord between these expectations and the realities of the URE was a reason why some students did not evaluate their experience positively. The success of student integration into an academic community is contingent on the degree to which they have opportunities to adopt the normative values of peers, faculty, and the institution (Krabacher, 2008). Such opportunities were abundant in some projects, affording consequential advantages including further employment and honors/graduate research supervision. Some projects, however, provided few networking opportunities, with students assigned to these projects largely directed to work from home, in a solitary learning environment. Given that good research is often the product of collaboration (Krabacher, 2008), UREs that require excessive independence can provide an impoverished learning experience for students. Psychology educators need to place a greater emphasis on providing avenues for students to develop professional networks during their undergraduate studies, which is likely to garner other collegial benefits including a greater sense of community within the institution (Bridgstock, 2016). UREs can serve this purpose. Interestingly, while supervision is an integral part of WIL and UREs in most learning institutions, it is an area that appears to be poorly understood and regulated. The mechanisms for monitoring the quality and consistency of supervision are not clearly defined, nor standardized from one situation to another (Lipp et al., 2007; Roberts and Seaman, 2018a). The limited oversight of the URE we studied resulted in an insufficient variety of experiences (and particularly social experiences) in some projects, and thus some students appeared to reap greater benefits than others. Perhaps there is a need to support the URE supervisors to provide an optimal experience for students, in a way that is increasingly recognized as important for dissertation supervisors (Roberts and Seaman, 2018a,b).

As evidenced by comments concerning expectations made by both faculty and student participants, the quality of a URE, and its potential to lead to a publication on which an undergraduate student is a co-author, is shaped by an interaction between content, supervision, other relationships, and student attitudes (Salm, 2015). As was clear with regard to the present URE program, providing money to involve students in research was not enough. Indeed, while the financial element of the URE program attracted participants, it did not necessarily provide space, teams, and supervisor training. These aspects appear to be integral to providing a high-quality experience—positive for both students and faculty—which could potentially result in additional outcomes, such as increased publication rates. Perhaps the selection process for admission to the scholarship program could include more information about topics and reveal the faculty running them, students could then filter their choices accordingly to tailor the experience to their strengths and preferences. Likewise, faculty could propose a list of project-specific desirable skills, which would enable the student to gain maximum benefit from the URE. Granted, more information may make the matching process more complicated to administer. However, it may also provide students with the choice to decline a project from the outset, if it is unlikely to meet their expectations. Interestingly, three of the students interviewed, who had positive experiences, spoke openly about having no interest in their topic in the beginning, but about enjoying it by the end. This shifting of interests has been documented in previous URE research, suggesting that students’ initial affinity with a topic is not critical to the success of a URE, although it can impact on student motivation in the early stages of a project (Krabacher, 2008). When considering the implications for developing future WIL and URE opportunities, if funding is prohibitive, most participants did express their willingness to participate in UREs as volunteers. The opportunities for professional development and the possibility of publication or conference presentations are sufficient motivation to become involved. However, such schemes run the risk of exposing keen students to exploitation and must be managed carefully.

When interpreting the results of this study, readers should keep in mind the usual caveats around small sample sizes and generalizability. The small sample size is vulnerable to homogeneity, with convenience sampling making it impossible to ensure diversity of demographics. Due to the competitive nature of the program, all students in the study were high achievers, and therefore, this study was unable to focus on how UREs can engage students with average grades. Furthermore, our study appears to have a responder bias in that we captured only those students who were studying at the university (either completing the undergraduate course or enrolled in graduate programs). We were not able to reach graduated students, due most likely to outdated contact details in university records. These issues reduce the extent to which this study can comment on the perceptions of students no longer affiliated with the university in some way.

In terms of future research, longitudinal tracking of the students could afford greater insights into how UREs can promote undergraduate publication. This is important to know so that those specific components or “key ingredients” of UREs that are more likely to facilitate publication can be encouraged. This topic lends itself to mixed methods research. Conducting surveys after every URE could enable quantification of key issues, with interviews to capture the nuances that surveys might overlook. Future research could also focus on devising mechanisms to monitor supervision quality, ensure that UREs afford a diversity of opportunities, and that they are accessible to a broader cross-section of students. Further, given that the program we have described has been discontinued due to funding cuts, voluntary schemes, which were recommended by both students and faculty, should be developed and studied.

This study contributes to existing literature on UREs in psychology by exploring factors that may promote and undermine their success and their ability to promote publication by undergraduate students in psychology. For most participants, the opportunities afforded by the program were positive and advantageous, confirming the raft of literature suggesting that UREs can make a significant contribution to outcome such as employability (Lipp et al., 2007; Cranney et al., 2008; Hamilton et al., 2018). Additionally, the experience of faculty and staff reported here provides guidance for future iterations of UREs in undergraduate psychology programs. In particular, publishing with undergraduates is possible but any such URE programs must be given careful thought to optimize student and faculty experiences and promote the likelihood of publication. The lessons we have learned suggest that the variability in quality of UREs depends on networking, supervision, access to teams versus isolation, resourcing, and matching of topics and people, which were all areas found to contribute toward either strong positive or strong negative outcomes. The value of UREs is well established outside of psychology, but there is a need to develop and administer them in a way that affords the opportunity to more students beyond the target demographic of only high achieving students so that more can benefit from the opportunity to contribute to and co-author publishable work from URE experiences.

## Ethics Statement

This study was carried out in accordance with the recommendations of the Human Research Ethics Committee at Curtin University with written informed consent from all subjects. All subjects gave written informed consent in accordance with the Declaration of Helsinki. The protocol was approved by the Human Research Ethics Committee at Curtin University.

## Author Contributions

This research was conducted by RG under the supervision of LB and PA. All four authors contributed to the design of the study. RG collected and analyzed the data with contributions from LB and PA. RG led the writing of the manuscript, with input from LB, AK, and PA.

### Conflict of Interest Statement

The authors declare that the research was conducted in the absence of any commercial or financial relationships that could be construed as a potential conflict of interest.
