# Supplementary material for: The Summer Undergraduate Research Experience as a Work-Integrated Learning Opportunity and Potential Pathway to Publication in Psychology
Source: Front Psychol. 2019 Mar 20;10:541. doi: 10.3389/fpsyg.2019.00541 (PMC6435955; doi:10.3389/fpsyg.2019.00541)
Supplement: Supplementary file 1 [file Table_1.DOCX]

Supplementary Material

Prior to each interview each participant was provided with a participant information statement, reminded of their rights, given the opportunity to ask questions, and asked to sign a consent form. Generic prompts used by the interviewer throughout each interview included:

- Can you tell me more about that?
- Can you elaborate on that?
- Could you give me an example of that?

At the conclusion of each interview each participant was thanked for their time.

# Interview Schedule for Student Participants

How would you describe your summer project?

- Why did you apply?
- What was it like?
- What did you learn?
- What did you expect versus what happened?
- Highlights/Lowlights/Memorable moments?
- What value would you assign to the experience?

How useful has your project been to what you are doing now?

- Are you able to elaborate?

Can you describe some of the skills you learned during this experience that you are still using today?

Could you tell me a bit about what you are doing for work since completing your project?

- Satisfying?
- As imagined?
- Linked to your project in any way?

Please describe any ways that your project has factored into professional relationships?

- Networking?
- Still in touch with supervisor?
- Work stemming from project?

What is your overall impression of your research project’s contribution to your career so far?

- Recommendations/Feedback?

Can you think of anything else that you would like to share about the Summer Scholarship Program that was not covered in the interview?

# Interview Schedule for Faculty Member Participants

Please tell me a bit about your time supervising in the Summer Scholarship Program.

- How much preparation does your involvement with the program require?
- Were there any moments that stand out?

Can you please tell me a bit about your teaching background and what led to your involvement with the Summer Scholarship Program?

- What were you hoping to gain from the experience?
- Can you assign a value to your participation?
- How many projects resulted in publication? Who was the lead author?

Can you tell me a bit about what you have been doing since the program finished?

- Anything similar?
- Describe how you engage with students now?

Please describe any teaching experiences with students of the program that stand out?

- Keep in touch and know their professional progress?

What is your overall impression of the Summer Scholarship Program’s contribution to the undergraduate psychology degree?

- Recommendations/Feedback?

Can you think of anything else that you would like to share about the Summer Scholarship Program that was not covered in the interview?
